# Optimal antenna locations of the VLBI Global Observing System for the estimation of Earth orientation parameters

**DOI:** 10.1186/s40623-020-01214-1

**Published:** 2020-06-18

**Authors:** Matthias Schartner, Johannes Böhm, Axel Nothnagel

**Affiliations:** 1 Department of Geodesy and Geoinformation, TU Wien, Wiedner Hauptstraße 8-10, 1040 Vienna, Austria; 2 TU Wien, Vienna, Austria

**Keywords:** Very Long Baseline Interferometry (VLBI), VLBI Global Observing System (VGOS), Global Geodetic Observing System (GGOS), Vienna VLBI and Satellite Software (VieVS), VieSched++

## Abstract

To support monitoring subtle effects in the Earth system such as a mean sea level rise of 3 mm/year, a next-generation VLBI system, the VLBI Global Observing System (VGOS), has been developed and a new VGOS station network is being built. However, the geometry of the current VGOS network and its planned extension suffer from a lack of stations in the southern hemisphere. In this investigation, we identify optimal locations for additional VGOS radio telescopes with a new method based on bulk observing schedule generation and subsequent large-scale Monte-Carlo simulations. The location of the additional station is varied over 477 possible locations, homogeneously distributed over land areas on the globe. For each antenna location, several schedules have been generated and simulated to minimize the effects of scheduling and the randomness of simulations. Thereby, it is possible to judge, in which regions an additional VGOS station would have the biggest impact on the precision of the estimated geodetic parameters, in our case assessed by the repeatabilities of the estimated Earth orientation parameters (EOPs). To generate highly optimized schedules and to remove effects due to non-optimized scheduling, a total of 93 thousand schedules were iteratively generated, investigating over 300 billion scans and 2.4 trillion observations. Each schedule was further simulated 1000 times, leading to over 5 trillion simulated and analyzed observations. Although the optimum location of a future VLBI station depends on the EOP of interest and the geometry of the existing network, it is shown that the more the VGOS network grows, the more the lack of southern stations becomes prominent. The best location for an additional VGOS station for most EOP components and especially in the case of future VGOS networks would be the southern part of South America. It is further shown that the location of the additional antenna highly determines the expectable precision of the EOP estimates. For a 6-station network, the location of an additional seventh antenna can improve the precision of the EOP by a factor of 2.4 to 3.8. For an 18-station network, the location of an additional 19th station still improves the repeatability by a factor of 1.6. It is also found that adding a station at some locations will not improve the precision at all.
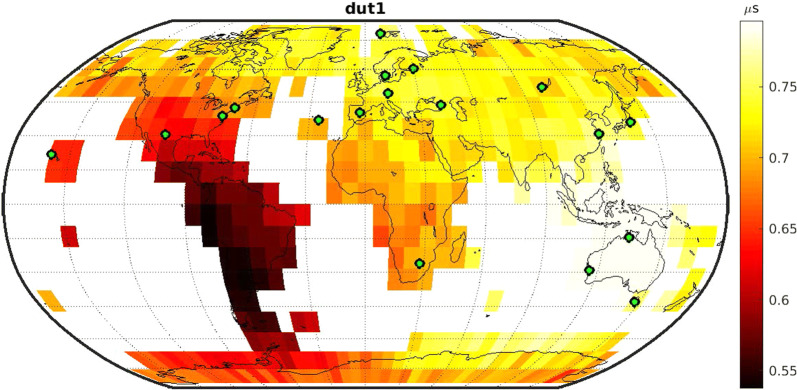

## Introduction

Very Long Baseline Interferometry (VLBI) measures the difference in arrival time of radio signals emitted from extragalactic radio sources at different telescopes by cross-correlation (Sovers et al. 1998). Therefore, it is necessary that multiple VLBI telescopes are composed in a so-called network to observe the same source simultaneously. Within a network, two VLBI telescopes are forming a baseline. The observation is the difference in arrival time between two telescopes each, thus one observation is made per baseline and per simultaneously observed radio source. By analyzing many of these VLBI observations together, parameters such as the orientation of the Earth in space or station coordinates and source positions can be estimated.

Multiple properties determine how accurate these geodetic parameters can be estimated, one of which is the geometry of the VLBI telescope network. For example, long baselines with east–west orientation are necessary for a precise measurement of the Earth rotation angle defined as the difference dUT1 between Universal Time (UT1) and Coordinated Universal Time (UTC). On the other hand, for a precise measurement of polar motion, long baselines with north–south orientation are beneficial (Dermanis and Mueller 1978). Although longer baselines lead to higher precision of Earth orientation parameters (EOP) theoretically, the selection of observations between telescopes forming a long baseline is significantly more difficult. The reason is that the commonly visible sky of these stations is very limited, which is diminishing the accuracy level again. Since different requirements exist for different geodetic parameters, it is a challenging task to define a good VLBI network for geodetic observations.

The Global Geodetic Observing System (GGOS) (Plag and Pearlman 2009; Beutler and Rummel 2012) defines accuracy requirements for monitoring global change, such as sea level rise (Blewitt et al. 2010). To reach these accuracy levels, a next-generation VLBI system called the VLBI Global Observing System (VGOS) has been developed (Petrachenko et al. 2009) and a new network of VGOS telescopes is being built. Choosing proper site locations for new VGOS telescopes has to consider multiple conditions, such as funding opportunities, radio frequency interference (RFI), co-locations to other space geodetic techniques, available infrastructure, security aspects, and also the expected benefit in terms of accuracy of geodetic parameters. Several studies were investigating possible new VGOS telescope locations in terms of their expected precision based on different approaches. For example, work by Hase and Pedreros (2014) used a most remote point method to iteratively define a homogeneous network based on Delaunay triangulation. Investigations by Pavlis et al. (2008) compared simulations of different network designs. A work by Merkowitz et al. (2018) uses satellite laser ranging (SLR) and VGOS VLBI simulations to plan the expansion of the NASA Space Geodesy Network, based on the projected accuracy of terrestrial reference frame origin, scale and orientation.

A commonly used approach to study VLBI networks is to add a new antenna to an existing network and compare simulation results. Examples for this approach can be found in several publications: Schartner et al. (2017) investigated the impacts of a new antenna in Africa based on this method. Kareinen et al. (2017) tested possible tag-along station locations for *Intensive* networks. Glaser et al. (2017) used a similar approach of adding antennas to existing global VLBI networks. The same concept was used by Kehm et al. (2019) for the identification of optimal locations for new SLR stations for GGOS.

In this work, we identify optimal locations for additional VGOS radio telescopes with a new method based on bulk observing schedule generation and subsequent large-scale Monte-Carlo simulations. We add a telescope to a real or already planned VGOS network as described in “Method” section, thereby assessing the deficiencies of the current and planned VGOS network. In total, 477 locations of possible new positions are tested, which are distributed homogeneously over the globe, see “Antenna locations” section. This study aims at investigating in which regions new VGOS stations would be most valuable for the determination of highly precise EOP. Since the simulations are based on observing plans, specifically generated for the tested configurations, special emphasis is given to providing optimal observation schedules because the observing plans can heavily impact the results as described in “Scheduling” and “Scheduling effects” sections. “Simulation” section summarizes how the simulations were made, while “Analysis” section describes how the simulations were analyzed to derive the geodetic parameters. The primary metric for the comparison of the best station location is the repeatability value of the EOP computed from the Monte-Carlo simulations. The results are summarized in “Results” section. Finally, “Conclusion” section concludes the results and “Outlook” section lists possible improvements for further studies.

## Method

The aim of this study is to determine optimal antenna locations for an additional telescope to be added to three example VGOS networks with 6, 12, and 18 antennas which represent the current status and expected evolution of the VGOS network (Behrend et al. 2019). Antennas from these networks are referred to as the “fixed antennas” and the networks as “fixed networks” in the following chapters.

To each of these fixed networks, one new additional antenna was added and it is referred to as the “variable antenna”. Therefore, the total number of antennas per network is 6 + 1, 12 + 1 and 18 + 1, where the first number stands for the number of fixed antennas and the + 1 refers to the variable antenna which was added to the fixed networks.

The antenna specifications of the fixed antennas needed for the scheduling task are based on the official IVS catalog files (Vandenberg 1997). For example, the slew speed of the AUSCOPE antennas (Lovell et al. 2013) was set to 300° per minute in azimuth and 75° per minute in elevation while most other telescopes were scheduled with slew speeds of 720° per minute in azimuth and 360° per minute in elevation. If an antenna was not included in the official IVS catalogs, it was assumed that its location is close to an existing VLBI antenna and the slew speed was set to 720° per minute in azimuth and 360° per minute in elevation. This was the case for fixed antennas that are currently under construction or planned as well as for every variable antenna. The fixed antennas forming the 6-station network are GGAO12M, KOKEE12M, ONSA13NE, RAEGYEB, WESTFORD, and WETTZ13S. For the 12-station network, the following antennas were added to the 6-station network: HOBART12, ISHIOKA, RAEGSMAR, and NYALE13S, as well as new VGOS stations near HART15M and SESHAN25. The 18-station network is extending the 12-station network with the antennas MACGO12M, KATH12M, and YARRA12M, as well as new VGOS stations near BADARY, SVETLOE, and ZELENCHK.

There were two main challenges for conducting this study: first, the global scale of the sample positions for the new telescope and second, optimizing the scheduling process for different network geometries require different strategies and approaches. Addressing both of these challenges requires a high number of schedules and simulations and thus leads to high computational costs.

**Fig. 1:**
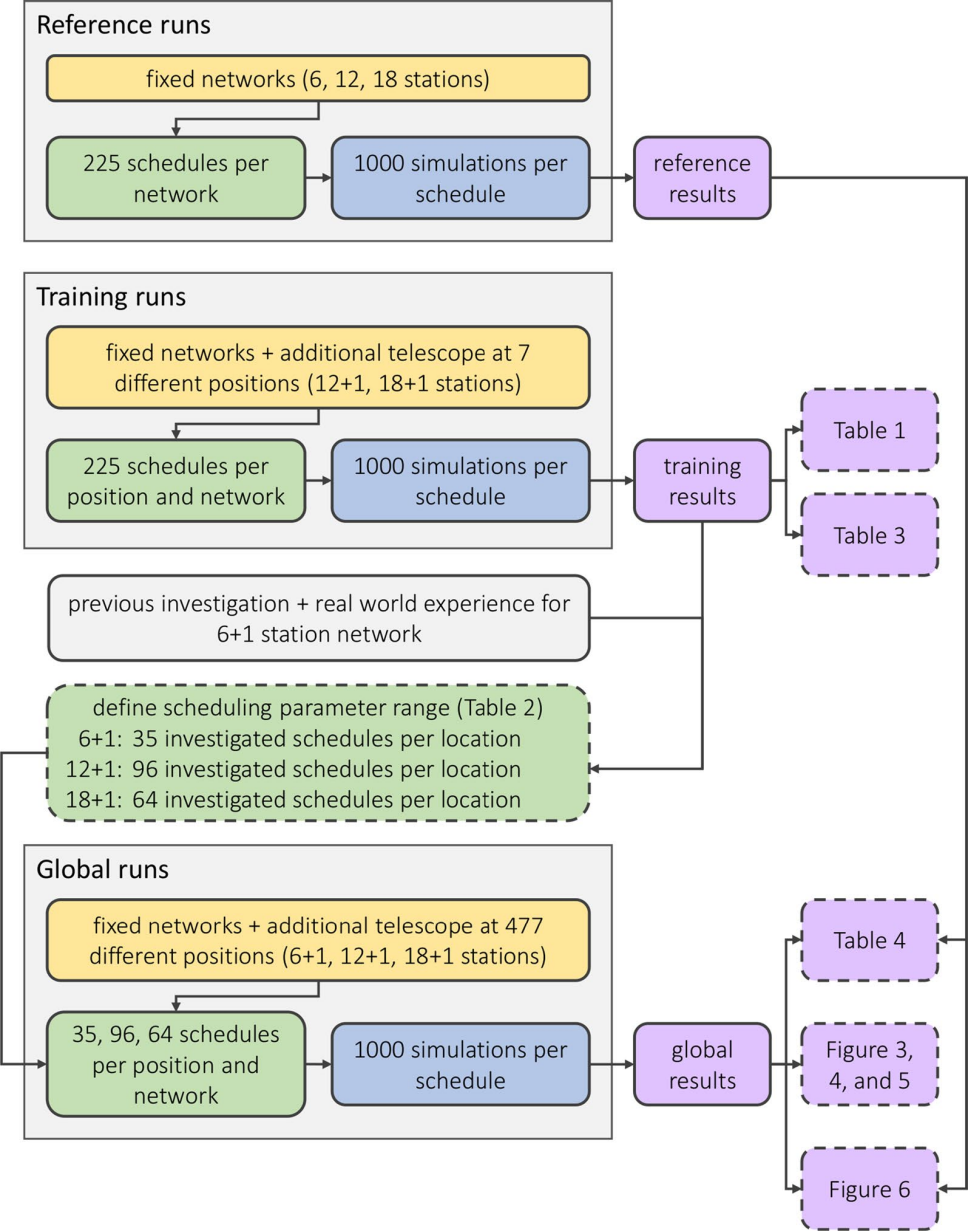
Flowchart of method used in this study. Orange boxes, network definitions; green boxes, scheduling-related information; blue boxes, simulation-related information; purple boxes, results. Dashed boxes highlight results visualized in tables and figures in this paper

**Fig. 2:**
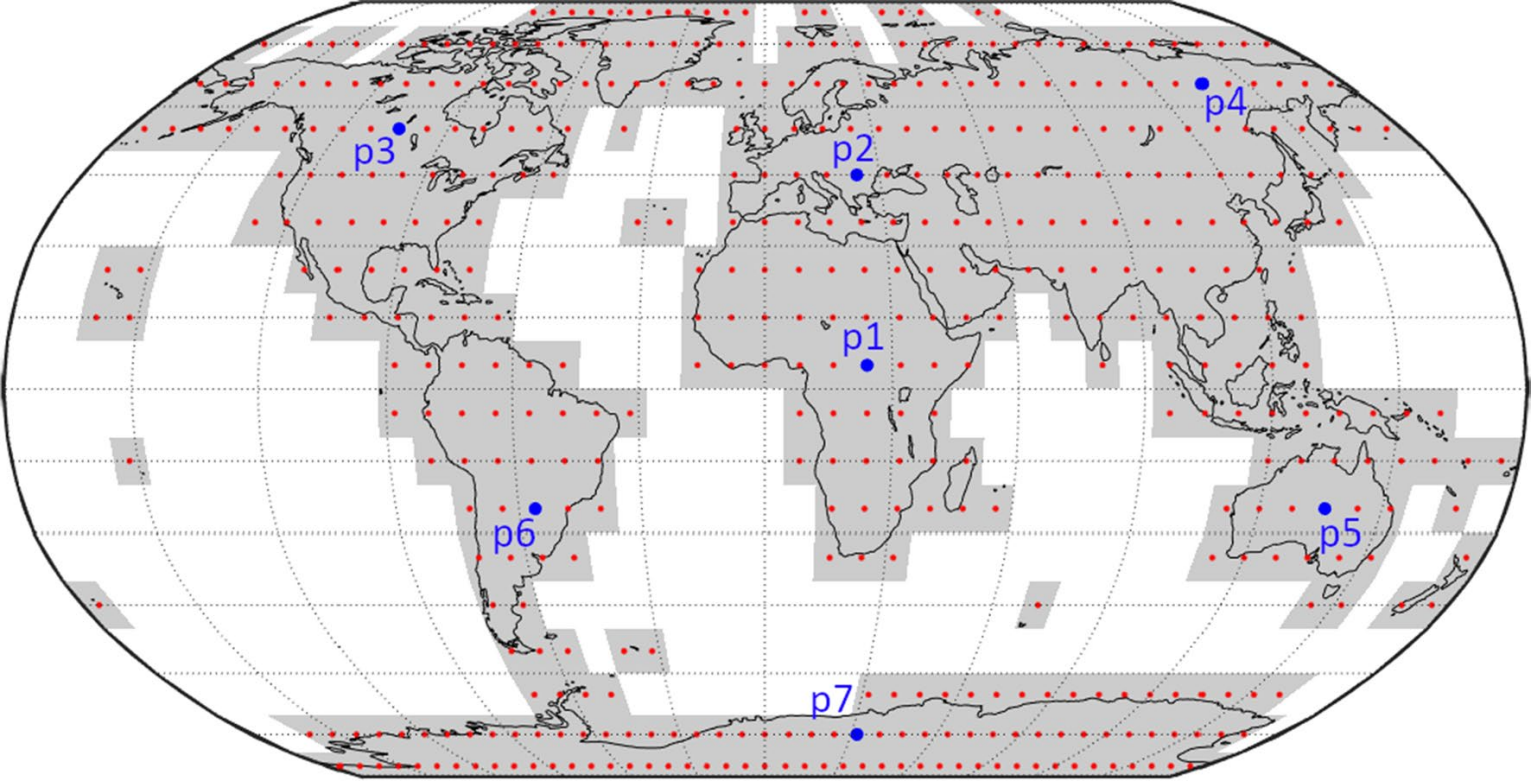
Investigated antenna positions (small red dots) and their corresponding grid cells (grey areas). The big blue dots highlight the seven training locations used to define the weights used in scheduling, see “Scheduling” section

To reduce the computational cost, the location of the variable antenna was limited to 477 possible positions, see “Antenna locations” section. For the same reason, additional steps of restricting the scheduling input parameter values (explained in “Scheduling” section) were introduced with seven globally distributed antenna locations being selected as “training locations” for the 12 + 1 and 18 + 1 networks (p1 to p7 in Fig. 2). For each training location and fixed network, a high number of schedules with a large range of scheduling input parameter values were generated and analyzed to determine which combination of scheduling input parameters was required to optimize the schedules of these training networks, see “Scheduling” section.

This training step was not explicitly done for the 6 + 1 station network for various reasons. The fixed 6-station network comprises antennas in Europe and North America only. Therefore, generating schedules for this network is easier compared to scheduling global networks. Also, this network was already investigated during a previous investigation Schartner and Böhm (2020) and for real experiments.

Based on these results, the total range of scheduling input parameter values was reduced, resulting in a lower number of necessary schedules for the 477 globally distributed variable antenna locations and fixed networks, see “Scheduling” section. Figure 1 displays a flowchart describing the method used in this work.

The “reference runs” were computed based on the fixed networks only without any additional antenna. The “training runs” were computed based on the fixed networks plus one additional antenna varied over the seven different training locations (p1 to p7) shown in Fig. 2. These networks are further referred to as the “training networks“ in the following. Both, the reference runs and the training runs were scheduled with a high number of 225 different schedules with a wide range of different scheduling input parameter values to investigate which parameter values are reasonable candidates for the full investigation of all antenna locations. Finally, the “global runs” were investigating the fixed networks plus one additional antenna varied over all 477 possible locations with the reduced number of schedules based on the results from the training runs, see “Scheduling” section.

### Antenna locations

The possible variable antenna locations were identified based on a simple regular grid with 10° spacing in latitude and 8° spacing in longitude over the main global land areas, resulting in 477 possible antenna positions as shown in Fig. 2. This leads to a total of $3\cdot 477=1431$ investigated networks. This discretization was chosen as being a good compromise between a high resolution and still reasonable computational cost. Every possible variable antenna location is marked with a red dot in Fig. 2. The grey areas depict the grid cells of all possible antenna locations.

The first step of our approach was to investigate training runs with the seven locations marked with blue dots (*p*1–*p*7). The locations were randomly chosen while providing a good test set with global coverage. This served to identify the range of the scheduling input parameter values, which were necessary to provide a high-quality schedule for every variable antenna location and for keeping the computational costs at a reasonable level (see “Scheduling” section).

The variable antenna height above sea level of all test positions was calculated based on a digital elevation model using the average elevation of the land areas within the grid cell of each location. The main reason for calculating the station height was to get realistic 3D telescope coordinates. However, the actual effect of antenna height component on scheduling is negligible.

### Scheduling

This study is based on assessing the expected EOP precision for different network geometries. Therefore, it is necessary to minimize effects which are caused by non-optimized scheduling. The difficulty arises since different network geometries require different scheduling input parameter values and approaches to generate an optimized schedule for the best possible result. Consequently, the schedule of every variable antenna location and network had to be individually optimized to provide a realistic number and distribution of observations and to be able to identify solely the effect of network geometries on EOP precision.

The optimization of the schedules can be achieved using VieSched++ (Schartner and Böhm 2019) and in particular its multi-scheduling feature. For geodetic VLBI scheduling and in particular for VGOS scheduling, a proper selection of the so-called weight factors (Schartner and Böhm 2019) is elementary for generating an optimized schedule (Schartner and Böhm 2020).

Several optimization criteria exist for generating a good geodetic schedule characterizing different scheduling approaches. In this study, the four most important optimization criteria and their corresponding weight factors $\omega$ were used, namely “*sky-coverage*” ($\omega _{{\text{sky}}}$), “*number of observations*” ($\omega _{{\text{nobs}}}$), “*duration*” ($\omega _{{\text{dur}}}$), and “*idle time*” ($\omega _{{\text{idle}}}$). The *sky-coverage* optimization criterion tries to distribute the observations with respect to azimuth and elevation at each station to provide an evenly distributed sky-coverage. This helps to estimate time delays caused by the wet part of the troposphere which is considered to be one of the dominant error sources in geodetic VLBI (Schuh and Böhm 2013). The *number of observations* optimization criterion tries to schedule as many observations as possible, which helps during the analysis by increasing the redundancy. The *duration* optimization criterion puts the focus on scans that finish their observation within a short time. Therefore, it covers not only the observation duration but also slew time, calibration time and overhead time needed to execute antenna control commands. Since the observing time of all scans is set to 30 s in this study (as it is the case for most current VGOS sessions) and calibration and overhead times are constant for all scans, this results in a situation where the *duration* optimization criterion prefers scans with a short slew time. The *idle time* optimization criterion is mostly used as a precaution to make sure that every station is observing regularly. It gradually increases the probability of scheduling scans containing stations that were previously not scheduled for a longer period (Schartner and Böhm 2019).

The weight factors $\omega$ define how much each optimization criterion contributes for to the scan selection (Schartner and Böhm 2019). One key aspect of optimized geodetic scheduling is to balance the different optimization criteria and thus scheduling approaches through the weight factors. A proper selection of weight factors is far from trivial since multiple optimization conditions are used with different requirements, which, in some cases, are conflicting with each other (Gipson 2010).

Previous studies (Schartner et al. 2017; Schartner and Böhm 2020) as well as experience with actually observed schedules indicate that significant improvements can be realized by optimizing scheduling through a proper balance of these four optimization criteria. One conceptually easy and at the same time very effective approach for finding proper weight factor values is to simply try out different weight factor combinations (based on a gridwise combination) and to base the decision on assessing the simulation results for identification of the best combination. The study for determining the necessary weight factor combinations was only done for the 12 + 1 and 18 + 1 training networks and not for the 6 + 1 network, see “Method” section above. This leads to a total of 14 training networks investigated in this study.

All four optimization criteria were used within the VieSched++ multi-scheduling feature with four possible values of $\{0.00$, 0.33, 0.67, $1.00 \}$. Using four equally spaced possible weight factor values provides a good compromise between computational cost and scheduling optimization.

This leads to 256 possible weight factor combinations $\vec {\omega _i}$:

1 $$\begin{aligned} \vec {\omega _i} = \begin{pmatrix} \omega _{{\text{sky}},i} \\ \omega _{{\text{nobs}},i} \\ \omega _{{\text{dur}},i} \\ \omega _{{\text{idle,}}i} \end{pmatrix} = \left\{ \begin{pmatrix} 0.00\\ 0.00\\ 0.00\\ 0.00 \end{pmatrix},\; \begin{pmatrix} 0.00\\ 0.00\\ 0.00\\ 0.33 \end{pmatrix},\; \begin{pmatrix} 0.00\\ 0.00\\ 0.00\\ 0.67 \end{pmatrix},\; \begin{pmatrix} 0.00\\ 0.00\\ 0.00\\ 1.00 \end{pmatrix},\; \begin{pmatrix} 0.00\\ 0.00\\ 0.33\\ 0.00 \end{pmatrix},\; \begin{pmatrix} 0.00\\ 0.00\\ 0.33\\ 0.33 \end{pmatrix}\dots \begin{pmatrix} 1.00\\ 1.00\\ 1.00\\ 1.00 \end{pmatrix} \right\} \end{aligned}$$

out of which 225 were further investigated since only the relative ratio of the weight factor values per combination is of importance, e.g., the following $\vec {\omega }$ would produce the same schedule and thus only one of these needed to be investigated:

2 $$\begin{aligned} \vec {\omega } = \begin{pmatrix} 0.00\\ 0.00\\ 0.00\\ 0.00 \end{pmatrix} \equiv \begin{pmatrix} 0.33\\ 0.33\\ 0.33\\ 0.33 \end{pmatrix} \equiv \begin{pmatrix} 0.67\\ 0.67\\ 0.67\\ 0.67 \end{pmatrix} \equiv \begin{pmatrix} 1.00\\ 1.00\\ 1.00\\ 1.00 \end{pmatrix}; \qquad \vec {\omega } = \begin{pmatrix} 0.00\\ 0.00\\ 0.00\\ 0.00 \end{pmatrix} \equiv \begin{pmatrix} 0.00\\ 0.00\\ 0.00\\ 0.33 \end{pmatrix} \equiv \begin{pmatrix} 0.00\\ 0.00\\ 0.00\\ 0.67 \end{pmatrix} \equiv \begin{pmatrix} 0.00\\ 0.00\\ 0.00\\ 1.00 \end{pmatrix}; \dots \end{aligned}$$

In contrast, using only three different weight factor values (e.g. 0.00, 0.50 and 1.00) instead of four, the total number of distinct weight factors’ combinations would drop to only 65 providing lower scheduling optimization while five different weight factor values (e.g. 0.00, 0.25, 0.50, 0.75 and 1.00) would result in 529 distinct weight factor combinations, thus increasing the computational cost with presumably little benefit in terms of scheduling optimization.

Each one of the 225 schedules per training network was simulated 1000 times and analyzed as described in “Simulation” and “Analysis” sections.

Based on the repeatability values of the estimated EOP derived from the Monte-Carlo result, the best schedule was identified for each of the seven tested antenna positions *p*1–*p*7. The selection of the best schedule was based on the lowest simulated EOP repeatabilities per schedule. The seven different weight factor combinations ($\vec {\omega }_{p1},\; \vec {\omega }_{p2},\; \vec {\omega }_{p3}\; \dots \; \vec {\omega }_{p7}$) leading to the best overall precision for the seven antenna test positions are listed in Table 1. We assume that they are a good representation of all possible scenarios on the globe. These seven combinations were used to identify the span of the weight factor values to be used for all other test locations.

**Table 1:** Weight factor combinations leading to the best schedule for the 12 + 1 and 18 + 1 station training networks

12 + 1	$\omega _{{\text{sky}}}$	$\omega _{{\text{nobs}}}$	$\omega _{{\text{dur}}}$	$\omega _{{\text{idle}}}$	18 + 1	$\omega _{{\text{sky}}}$	$\omega _{{\text{nobs}}}$	$\omega _{{\text{dur}}}$	$\omega _{{\text{idle}}}$
$\vec {\omega }_{p1}$	0.67	1.00	1.00	0.33	$\vec {\omega }_{p1}$	0.67	0.00	0.33	0.67
$\vec {\omega }_{p2}$	1.00	0.67	0.33	0.33	$\vec {\omega }_{p2}$	1.00	0.00	0.67	1.00
$\vec {\omega }_{p3}$	0.67	1.00	0.67	0.33	$\vec {\omega }_{p3}$	0.33	0.00	0.33	1.00
$\vec {\omega }_{p4}$	0.33	0.67	0.67	0.33	$\vec {\omega }_{p4}$	1.00	0.33	0.33	1.00
$\vec {\omega }_{p5}$	0.67	0.33	0.67	0.67	$\vec {\omega }_{p5}$	1.00	0.33	0.67	1.00
$\vec {\omega }_{p6}$	0.67	0.00	0.67	1.00	$\vec {\omega }_{p6}$	0.33	0.00	0.00	0.00
$\vec {\omega }_{p7}$	1.00	0.33	0.67	0.67	$\vec {\omega }_{p7}$	0.67	0.00	0.00	0.33

Each row corresponds to one combination of weight factors $\vec {\omega }$ leading to the best result for a network with the additional antenna located at position *p*1–*p*7 (see Fig. 2)

For example, the necessary span for the $\omega _{{\text{sky}}}$ parameter for the 18+1 station network is calculated based on the seven weight factor parameter values $\omega _{{\text{sky}},p1},\; \omega _{{\text{sky}},p2},\; \omega _{{\text{sky}},p3}\; \dots \; \omega _{{\text{sky}},p7}$. The span is defined as the minimum and maximum of these values. For the 18 + 1 network and the $\omega _{{\text{sky}}}$ parameter, the resulting span is 0.33–1.00, see Table 2. We assume that using a gridwise combination of the weight factor values $\omega$ listed in Table 2, the resulting weight factor combinations $\vec {\omega }$ will provide reasonably well-optimized schedules for the remaining 470 variable antenna locations during the global runs.

**Table 2:** Table of weight factors identified from training runs (and earlier investigations of 6 + 1 network) for application in global runs

Weight factor	6 + 1 network	12 + 1 network	18 + 1 network
$\omega _{{\text{sky}}}$	$\{0.67,\;1.00 \}$	$\{0.33,\;0.67,\;1.00 \}$	$\{0.33,\;0.67,\;1.00 \}$
$\omega _{{\text{nobs}}}$	$\{0.33,\;0.67,\;1.00 \}$	$\{0.00,\;0.33,\;0.67,\;1.00 \}$	$\{0.00,\;0.33\}$
$\omega _{{\text{dur}}}$	$\{0.67,\;1.00 \}$	$\{0.33,\;0.67,\;1.00 \}$	$\{0.00,\;0.33,\;0.67\}$
$\omega _{{\text{idle}}}$	$\{0.00,\;0.5,\;1.00 \}$	$\{0.33,\;0.67,\;1.00 \}$	$\{0.00,\;0.33,\;0.67,\;1.00 \}$

As noted, the weight factor combinations $\vec {\omega }$ used during the global runs were gridwise combinations of the possible weight factor values. Similarly to the training runs, only the relative ratio of the weight factors is of importance, thus some weight factor combinations would lead to identical schedules and could be ignored. The total number of weight factor combinations and thus schedules per variable antenna location was 35 for the 6 + 1 networks, 104 for the 12 + 1 networks and 66 for the 18 + 1 networks. For performance reasons only 96 of the 104 schedules per antenna location for the 12 + 1 networks and only 64 of the 66 schedules per antenna location for the 18 + 1 networks were generated, while making sure that the combinations listed in Table 1 were still represented.

Since the weight factor values for the 6 + 1 networks were chosen based on previous investigations and real world experience (see “Method” section), the value of the idle time weight factor $\omega _{{\text{idle}}}$ for the 6 + 1 station network in Table 2 was set to 0.5 instead of a multiple of 0.33 as done for all other parameters. This is not a problem for this study since the idle time weight factor is mainly used to ensure that every station is regularly observing, which should generally be the case for fast slewing VGOS stations with an observing time of only 30 s.

The total number of schedules generated for the training runs was 3150, with 1575 ($7 \cdot 225$) schedules for the 12 + 1 networks and 1575 ($7 \cdot 225$) schedules for the 18 + 1 networks. The total number of generated schedules for the global runs was 93,015, with 16,695 ($477 \cdot 35$) schedules generated for the 6 + 1 networks, 45,792 ($477 \cdot 96$) schedules for the 12+1 networks, and 30,528 ($477 \cdot 64$) schedules for the 18+1 network.

Every schedule was generated by allowing subnetting (Gipson 2010) and using an iterative source selection (Schartner and Böhm 2019) with a minimum number of three scans per source. The iterative source selection requires that multiple schedules have to be generated iteratively to finalize the source selection for the final schedule. Therefore, the total number of generated schedules for the global runs was in fact 364,502, out of which the 93,015 final schedules were extracted. In total, more than $3.28\times 10^{11}$ scans and $2.14\times 10^{12}$ observations were considered by VieSched++ during the generation of the schedules.

### Scheduling effects

This section discusses why individually optimized weight factor combinations are necessary for a global investigation of network geometries and why it is not sufficient to simply use one set of average weight factors for all networks. The demonstration is based on the simulated dUT1 repeatability from the seven 18 + 1 training networks. However, it is to note that the same applies for all EOP and all networks. Table 3 compares the results for the simulated dUT1 repeatability using different networks and weight factor combinations. Each column represents one training network with the variable antenna located at the training position visualized in Fig. 2 and each row one set of weight factor combinations $\vec {\omega }$. The first row $\vec {\omega }_{p1,{\text{dUT}}1}$ refers to the weight factor combination $\vec {\omega }$ leading to the best simulated dUT1 repeatability for the 18 + 1 training network including the variable antenna at position *p*1, the second row $\vec {\omega }_{p2,{\text{dUT}}1}$ to the best weight factor combination $\vec {\omega }$ for an antenna located at position *p*2, and so on.

The cells of Table 3 show the simulated dUT1 repeatability from the seven training networks using the seven weight factor combinations $\vec {\omega }_{p1,{\text{dUT}}1}$ to $\vec {\omega }_{p7,{\text{dUT}}1}$ divided by the best simulated dUT1 repeatability of this network. This means that in column *p*1 every value is divided by the value of row $\vec {\omega }_{p1,{\text{dUT}}1}$, in column *p*2 every value is divided by the value of row $\vec {\omega }_{p2,{\text{dUT}}1}$ and so on. Therefore, the main diagonal in Table 3 lists all 1.00.

**Table 3:** Normalized dUT1 repeatability for the 18 + 1 network showing the importance of individually optimized weight factor combinations for each network geometry

Best parameters	Training antenna position						
	*p*1	*p*2	*p*3	*p*4	*p*5	*p*6	*p*7
$\vec {\omega }_{p1,{\text{dUT}}1}$	1.00	1.17	1.26	1.19	1.03	1.25	1.46
$\vec {\omega }_{p2,{\text{dUT}}1}$	1.12	1.00	1.36	1.08	1.12	1.23	1.28
$\vec {\omega }_{p3,{\text{dUT}}1}$	1.10	1.71	1.00	1.21	1.21	1.26	1.15
$\vec {\omega }_{p4,{\text{dUT}}1}$	1.42	1.38	1.19	1.00	1.19	1.23	1.22
$\vec {\omega }_{p5,{\text{dUT}}1}$	1.57	1.36	1.30	1.37	1.00	1.66	1.37
$\vec {\omega }_{p6,{\text{dUT}}1}$	1.40	1.34	1.48	1.37	1.28	1.00	1.04
$\vec {\omega }_{p7,{\text{dUT}}1}$	1.09	1.38	1.21	1.03	1.38	1.09	1.00

Similar results can be seen for the 6 + 1 and 12 + 1 networks and all EOP. Values larger than 1.00 indicate how much the results degrade when not using the optimum weighting scheme for a particular training antenna position

The values of each column (*pi*) list how much worse the simulated repeatability would become, if the preferred weight factor combination from a different variable antenna location ($\vec {\omega }_{pj,{\text{dUT}}1}, \; j \ne i$) would have been used instead of the weight factor combination working best for this variable antenna location ($\vec {\omega }_{pi,{\text{dUT}}1}$). The values of each row list the degradation of the simulated repeatability if the weight factor combination ($\vec {\omega }_{pi,{\text{dUT}}1}$) of one variable antenna location (*pi*) would be used for a different variable antenna location ($pj,\; j \ne i$) instead of the weight factor combination working best for this variable antenna location ($\vec {\omega }_{pj,{\text{dUT}}1}$). Therefore, Table 3 highlights the impact of non-optimized scheduling on the result. It is shown that the negative effect can be as large as $71\%$ (factor 1.71). This is especially noteworthy since only weight factor combinations which lead to the best result on one of the training networks were investigated. The effect would be even larger if any weight factor combination would have been used that is not that highly optimized for this session type and thus would lead to a poorer schedule in general.

To conclude this section with an example, we assume that the weight factor combination which leads to the best results for the training network *p*1, $\vec {\omega }_{p1,{\text{dUT}}1}$ (first row) had been used for scheduling the network including the variable antenna location *p*7. Then, the estimated precision of dUT1 for the training network *p*7 (last column) would be $46\%$ worse compared to the lowest repeatability gained using the best weight factor combination for this training network $\vec {\omega }_{p7,{\text{dUT}}1}$ (last column, last row). In this case, the degradation due to non-optimized scheduling would be as large as $46\%$ in terms of simulated dUT1 repeatability and thus obscuring the influence based on the different network geometries. This highlights how important proper scheduling and individually optimized schedules are for simulation studies in general and for this study in particular.

As already noted, although this section focuses on the effect on dUT1 for the 18 + 1 network, similar effects are seen for all EOP and all networks (not shown here).

### Simulation

Every schedule for each configuration was simulated 1000 times using VieVS (Böhm et al. 2018), leading to a total of over $9.3\times 10^7$ simulated datasets with $2.05\times 10^{11}$ simulated scans and $5.13\times 10^{12}$ simulated observations. The simulations include modeling of tropospheric delays, clock drifts, and white noise, similar as done in previous studies, such as those described by Pany et al. (2011) and by Petrachenko et al. (2009). The tropospheric delay was simulated using a constant tropospheric refractive index structure constant $C_n$ of $2.0\times 10^{-7} {\text{m}}^{-1/3}$ with a scale height of 2000 m (Nilsson et al. 2007) at all stations. The wind velocity was set to 8 m/s toward east. Clock drifts were simulated using the sum of random walk and integrated random walk (Herring et al. 1990) with an Allan Standard Deviation of $1\times 10^{14}$ s after 50 min. The white noise contribution was set to 4 picoseconds according to Petrachenko et al. (2009). No source structure effects were simulated since it is assumed that the source structure effects will be handled and corrected earlier during fringe-fitting, as for example also ionospheric effects. In addition, this study aims at finding the best station position and thus source-based effects such as source structure are assumed to play a minor role here. However, since source structure is assumed to heavily influence the geodetic results for VGOS (Anderson and Xu 2018), it is to note that the general scheduling logic of VGOS sessions might be adjusted in the future, in case imaging plays a more important role and becomes a dedicated goal of VGOS sessions. This possibility and its implications are kept aside for further studies.

### Analysis

The analysis of the 93 million simulated sessions with its over 5 trillion observations was done in VieVS with every simulated session analyzed individually. The analysis used a least-squares method as described in Schuh and Böhm (2013). The tropospheric wet delay was estimated every 15 min as piecewise linear offsets (PLO) with loose constraints of 1.5 cm. In addition, tropospheric gradients were estimated every 30 min as PLO with loose relative and absolute constraints of 0.05 cm and 0.1 cm. The EOP were estimated as daily PLO with very tight constraints of 0.1 $\upmu$as. These daily PLO with very tight relative constraints correspond to one estimated EOP offset per session. The clocks were estimated as a rate and quadratic term per session plus PLO every 60 min with loose constraints of 1.3 cm. One reference clock was kept fixed. Finally, station coordinates were estimated per session including a no-net rotation (NNR) and no-net translation (NNT) condition over all stations.

Repeatability values of the estimated EOP were calculated from the individual results of the 1000 simulations per schedule. Throughout this work, repeatability values were used as the metric to compare the different schedules and the different network geometries. It would have also been possible to use the formal uncertainties from the least-squares adjustment directly to compare the different results. However, it was assumed that the repeatabilities were more realistic to the extent that the simulation model of the delay errors (tropospheric turbulence, expected clock errors, and observation noise) gives a reasonable accounting for these effects. The drawback of using repeatability values is that a relatively high number of simulations are necessary to achieve reliable results.

## Results

**Fig. 3:**
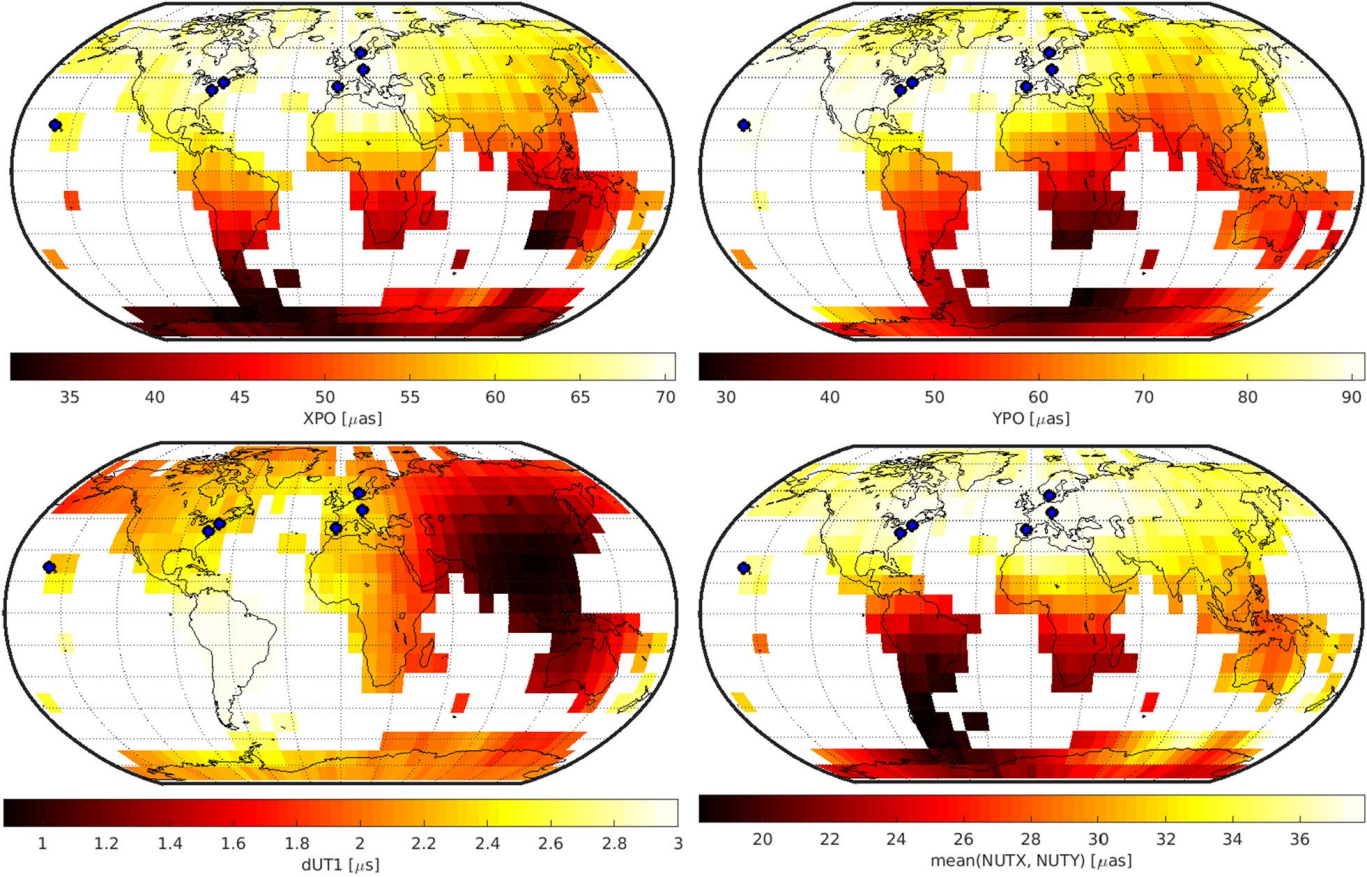
Precision of geodetic parameters for the 6 + 1 network. The fixed station network antennas are displayed by blue dots

**Fig. 4:**
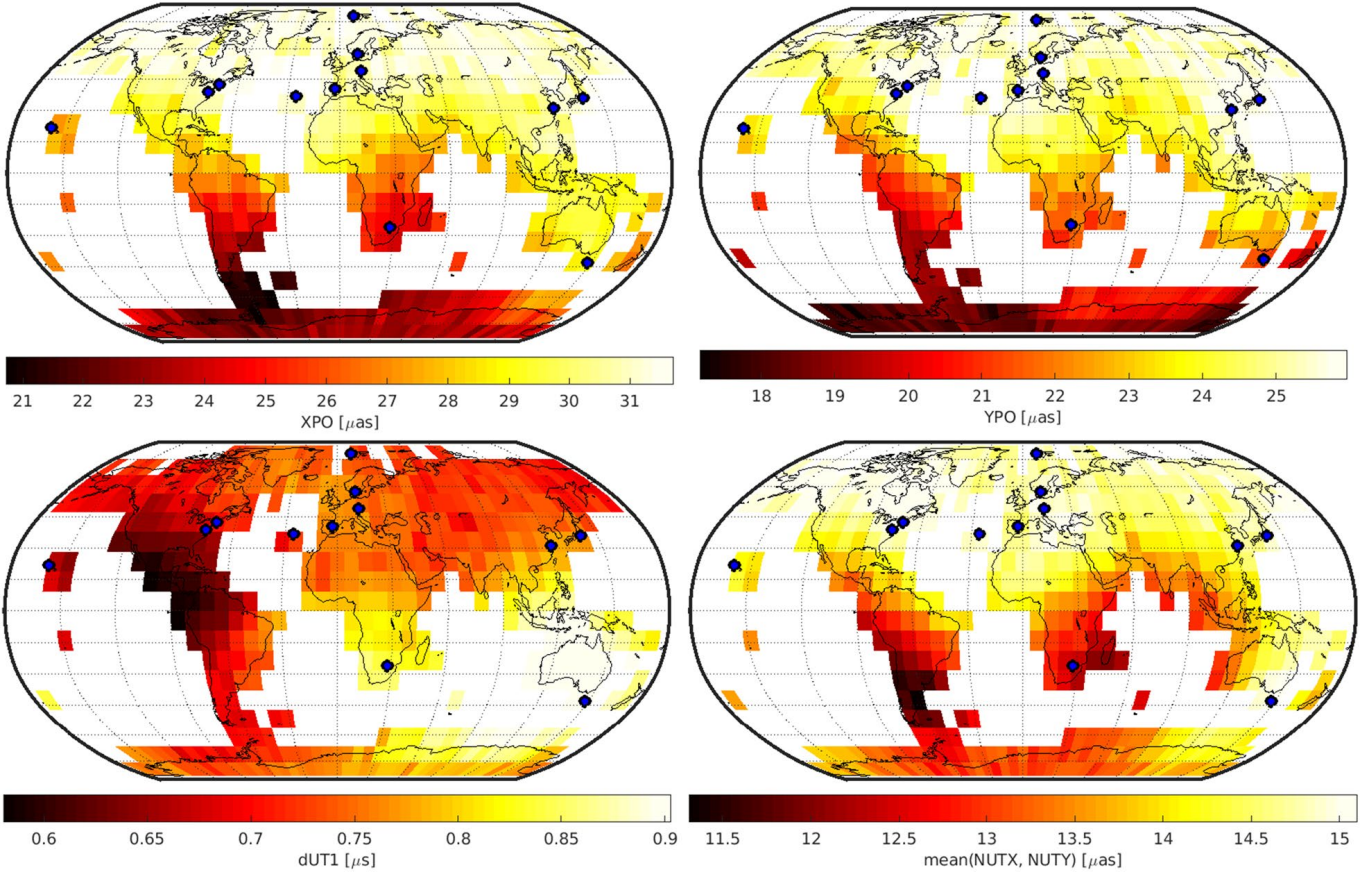
Precision of geodetic parameters for the 12 + 1 network. The fixed station network antennas are displayed by blue dots

**Fig. 5:**
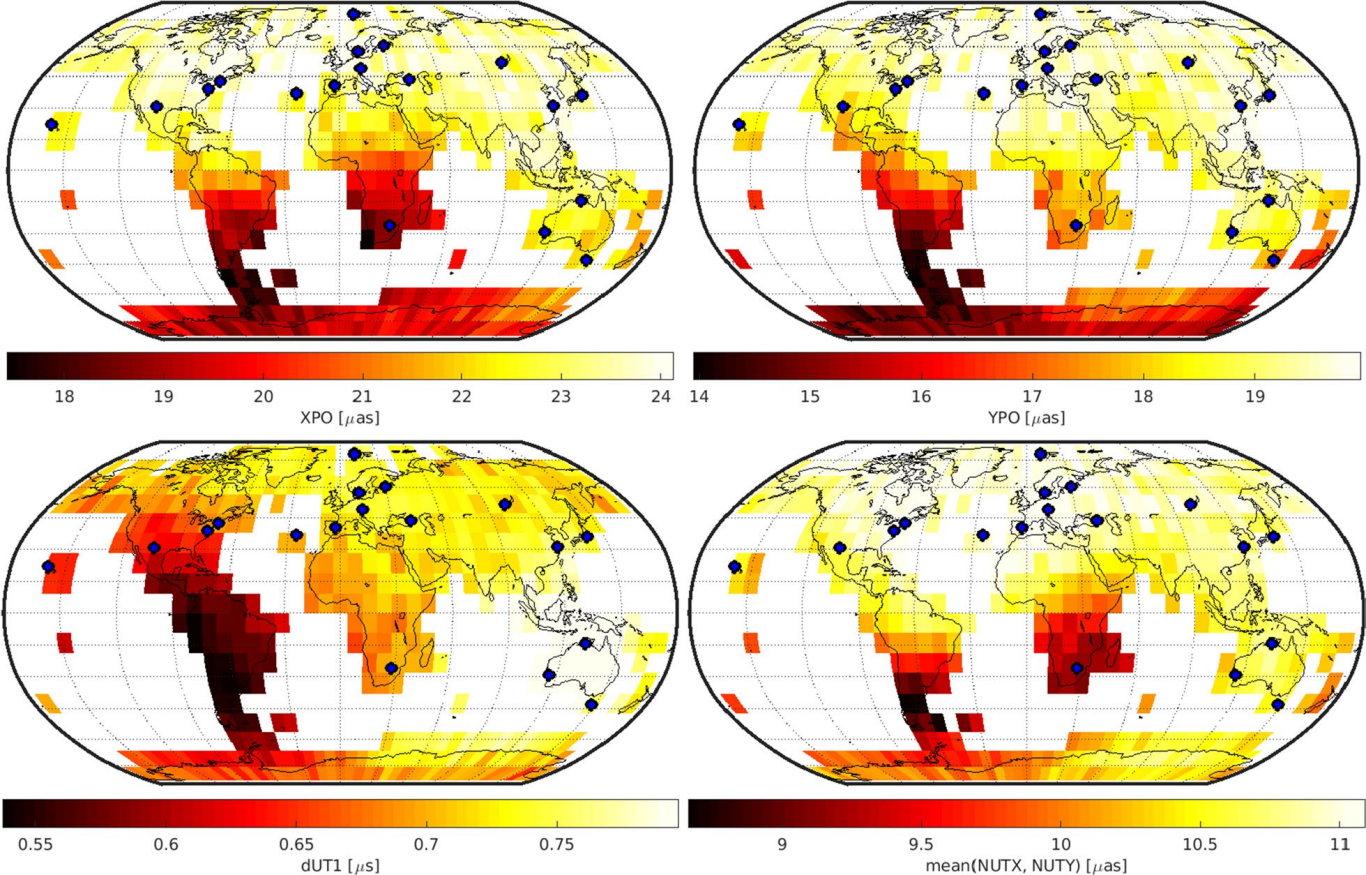
Precision of geodetic parameters for the 18 + 1 network. The fixed station network antennas are displayed by blue dots

Figures 3, 4, and 5 depict the geodetic results of the individual networks. In particular, they visualize the expected precision of the five EOP, namely polar motion in x- and y-direction (XPO, YPO), nutation in x- and y-direction (NUTX, NUTY) and the Earth rotation angle, expressed by dUT1. The precision is described by the repeatability of the 1000 simulation per schedule. The first row of Figs. 3, 4, and 5 displays the polar motion repeatability in the x-direction (left) and y-direction (right). The second row displays the dUT1 repeatability (left) and the average of the two nutation parameters (right). Since both nutation parameters yield almost identical results, the average of both values is visualized instead of adding a fifth plot showing each result individually. The fixed 6-, 12-, or 18-station network antennas are highlighted by a blue dot.

Every possible variable antenna location is represented by one cell as shown in Fig. 2 covering the grid cell of this location. The color code represents the best repeatability value gathered from the 1000 simulations of the different individual schedules. This means that every cell is represented by a color code that stands for the best schedule generated using a network consisting of the fixed antennas shown as blue dots and the variable antenna located in the center of the cell. This is a valid approach since in reality, the best schedule identified through the simulations will be chosen for the actual observations.

### 6 + 1 solution

Figure 3 depicts the results for the 6 + 1 network. For polar motion and nutation, clear minima can be identified in the southern hemisphere. In x-direction of polar motion, two minima can be seen which are roughly 180° apart in longitude. One is in the southern part of South America, while the other is in the south-west of Australia. In y-direction, only one minimum is visible which is located in and south of South Africa, directly in between the two minima in the x-direction. The minima can be explained by the additional long north–south baselines gained by adding a station in the southern hemisphere.

For nutation, both components can be estimated best by adding an antenna in the southern part of South America.

In contrast, dUT1 estimates show the best precision if an antenna would be added in south-east Asia. This minimum can be explained by the additional long east–west baselines which are most sensitive to dUT1.

Therefore, it is shown that the optimal antenna location depends on the parameter of interest and highlights the difficulty of a proper network definition for geodetic VLBI since different parameters have different requirements such as north–south or east–west baselines which lead to very different requirements for the network.

In general, the estimated EOP repeatabilities cover a broad spectrum depending on the location of the variable antenna. Therefore, this location defines the gain in precision to be realized by the new telescope.

### 12 + 1 solution

Figure 4 depicts the results for the 12 + 1 network. For polar motion and nutation, southern antenna locations lead to the best precision improvements. For polar motion, South America, the south of Africa and especially Antarctica would be the preferred antenna location. For nutation, South America and the area around the Indian Ocean including the southern part of Africa would lead to the best result. Surprisingly, the best location for dUT1 is achieved by putting the variable antenna in Central America. This location might be explained by the Hawaii–Central America–South Africa–Australia east–west connection mostly located at the southern hemisphere which serves as a counterpart to the existing northern hemisphere east–west connections, although it is not immediately obvious. Considering the composite of all EOP estimates, an antenna located in South America as far south as possible would be the best choice providing the best overall performance.

The overall EOP precision level of the 12 + 1 station network is a lot better compared to the 6 + 1 network solutions, see “Comparison of test setups” section, especially because the fixed 6-station network only consists of stations located in Europe and North America and is therefore not that sensitive for estimating all EOP components with the highest precision. In addition, the spectrum of the repeatability values is a lot broader in the 6 + 1 network solutions compared to those of the 12 + 1 network for the same reason. Since the fixed 6-station network is regionally restricted, the addition of a variable antenna at a remote location is a lot more impacting.

### 18 + 1 solution

Figure 5 depicts the results for the 18 + 1 network. In this case, the best location for improving all EOP is again in South America. For polar motion in x-direction as well as for the nutation parameters, putting the variable antenna in the southern part of Africa also leads to a considerable improvement in precision. In addition, parts of Antarctica are also beneficial target areas for a new telescope for polar motion estimates.

### Comparison of test setups

**Fig. 6:**
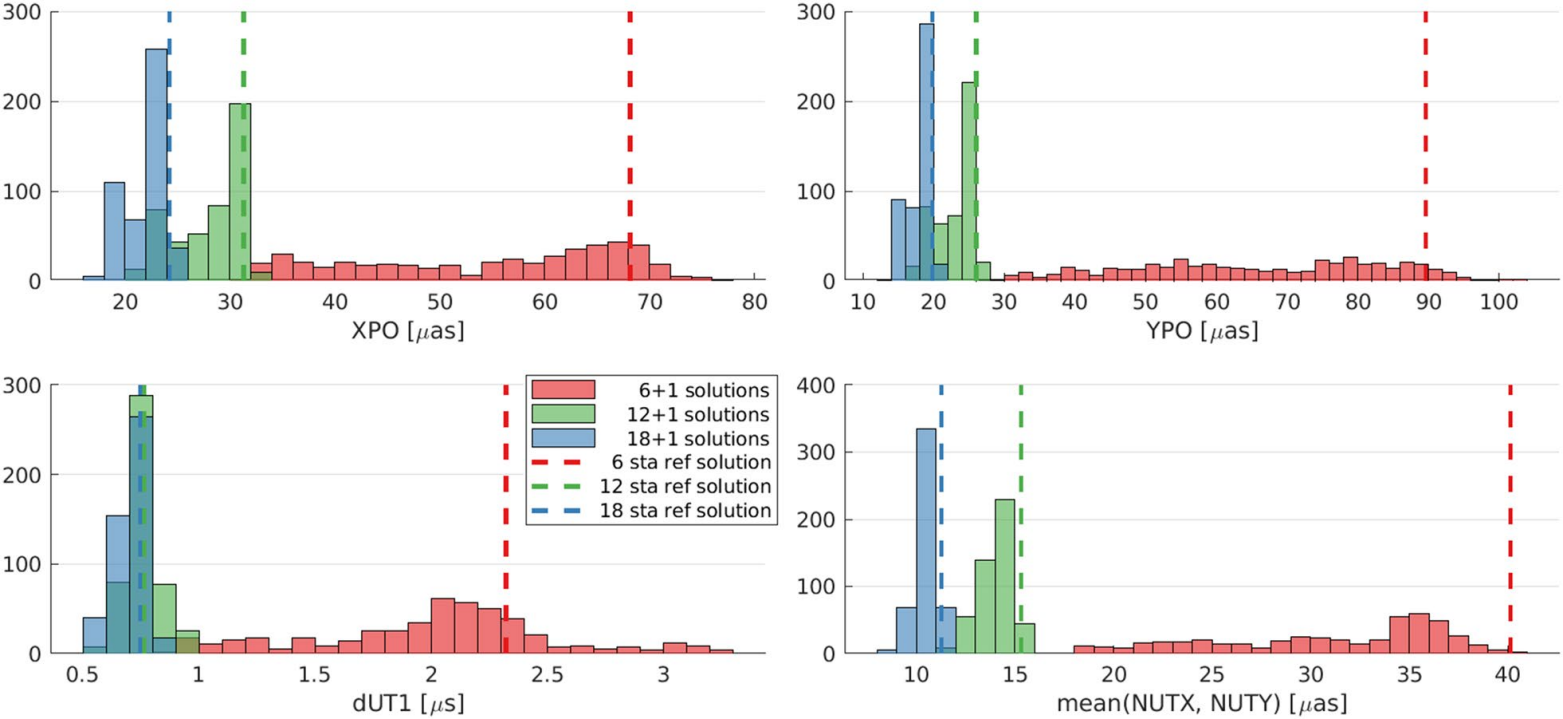
Histogram over the expected repeatability values visualized in Figs. 3, 4, and 5. The dashed vertical line indicates the simulated EOP repeatabilities of the fixed networks without an additional variable antenna

In a joint representation, the results of the three test setups (6 + 1, 12 + 1, and 18 + 1) can be discussed comparatively. Figure 6 compares the results of Figs. 3, 4, and 5 in terms of achieved precision. The histograms visualize the best repeatability values per variable antenna location. Consequently, each histogram contains 477 values. The results achieved from using only the fixed networks without an additional variable antenna are displayed by a dashed line.

By comparing the results of the individual 6 + 1, 12 + 1 and 18 + 1 station networks per EOP, it is not unexpected that we see bigger networks leading to better precision. Especially between the 6 + 1 and 12 + 1 station networks, big differences in distribution and level of precision are evident.

Furthermore, the histogram reveals that the addition of the variable antenna does not necessarily lead to an improvement in all cases. In some scenarios, the solution is better by just using the fixed network. The reason for this has to be investigated further. One explanation could be that if the variable antenna is in an unfavorable location, it will influence the schedule negatively by requiring more subnetting and increased idle times of telescopes. However, it is to note that even if the expected precision does not lead to an improvement in some regions, it might still be beneficial to build an antenna there to provide redundancy in case other stations are under maintenance.

The distribution of the histogram reveals how much the parameter depends on the network geometry. For the 6 + 1 station network, a broad spectrum of repeatability values is present. This means that the distribution of the results is highly dependent on the location of the variable antenna. In contrast, the spectrum of the 12 + 1 and 18 + 1 station networks is a lot narrower, meaning that the locations of the variable antenna do not influence the results as much.

**Table 4:** Minimum and maximum simulated EOP repeatability over all variable antenna locations, as well as their range and ratio in $\upmu$as and $\upmu$s, respectively

	Min	Max	Range	Ratio	Ref
6 + 1					
XPO	31	76	45	2.4	68
YPO	27	104	76	3.8	90
dUT1	0.9	3.3	2.4	3.7	2.3
NUT	18	41	23	2.2	40
12 + 1					
XPO	21	32	12	1.6	31
YPO	17	27	10	1.6	26
dUT1	0.58	0.98	0.40	1.7	0.76
NUT	11	16	4	1.4	15
18 + 1					
XPO	17	25	7	1.4	24
YPO	14	20	6	1.5	20
dUT1	0.54	0.84	0.30	1.6	0.75
NUT	9	11	3	1.3	11

Column “ref” lists the EOP repeatability value simulated using the fixed networks without any additional variable antenna

For further interpretations, we look at the dependency of the investigated EOP on the network geometry (Table 4). It lists the best and worst repeatability values, as well as their range, their ratio (reference is the best repeatability), and reference result from the fixed network alone. The higher the ratio, the more the precision is dependent on the variable antenna location. An example is the repeatability of polar motion in the y-direction for the 6 + 1 network solution that has a spread between 27 and 104 $\upmu {as}$ with a factor of 3.8 depending on the location of the seventh antenna. The higher the total number of stations, the lower the impact of the additional antenna becomes. However, even for the 18 + 1 network solution, the choice of a suitable location of the variable antenna can still improve the result by a factor of 1.6 compared to just choosing an arbitrary bad location.

## Conclusion

We described a new method for identifying the optimal position for an additional VGOS radio telescope based on bulk scheduling and massive Monte-Carlo simulations. As an immediate result, we present global maps of optimal locations depending on the configuration of the initial network and the target parameters to be improved. The maps can be interpreted easily geometrically for the 6-station base network covering only a limited area of the global sphere. However, for the larger networks interpretation becomes increasingly complex and the method reveals its potential for being an ideal tool for decision makers.

In general, the results of this study highlight the lack of southern stations for future VGOS network constellations. Adding an antenna in the southern hemisphere leads to better results for almost all EOP. Based on the simulations presented in this study, the best location for a new VGOS antenna would be the southern parts of South America. One appropriate new VGOS candidate site could be the position of the existing AGGO VLBI station in Argentina since some of the necessary infrastructure is already available and the simulated repeatabilities show reasonable high precision in this area.

Furthermore, it is shown that the location of one single additional antenna can significantly improve the EOP precision, even for bigger networks. For the 6-station network, the optimal choice of a position for an additional antenna compared to an arbitrary location affects the precision by a factor of 2.2 to 3.8 depending on the EOP component. For the 12-station network, the effect is between 1.4 and 1.7 and for the 18-station network, it is still between 1.3 and 1.6.

In addition, this study confirms the importance of proper scheduling for this kind of simulation study. It is shown that it is not sufficient to simply use the same scheduling parameters for all network geometries since the impact of non-optimized scheduling is on the same order of magnitude as the impact of the network geometry. Therefore, it would be best if all further simulation studies account for the effect of scheduling in their work.

For real-life decisions, of course several additional constraints need to be considered besides the accuracy information of the derived geodetic parameters for the selection of new VGOS station locations. Important are also funding possibilities, RFI, available infrastructure, security aspects, and other operational requirements.

## Outlook

Although this study provides very sophisticated and high-quality results, especially through eliminating effects caused by scheduling and using state-of-the-art simulation approaches, several improvements might be possible. The simulation could be improved using individual tropospheric turbulence parameters per antenna location based on the climatic situation, as for example done by Kareinen et al. (2017). In our study presented here, the troposphere is assumed to be the same for all antennas using the same turbulence parameters independently of the antenna location, although the average troposphere for antennas located in the rainforest is different than for antennas located in the dessert or for antennas located in the Arctic regions. However, the selection of realistic turbulence parameters all over the world is not trivial since it is very variable and changes over short periods. Therefore, using the same parameters is considered a good first guess with the added benefit, that the results are only dependent on the network geometry and not on the climatic properties of the regions.

In addition, this study focuses on geodetic parameters, mainly the EOP precision expressed through the repeatability values over 1000 simulations. It is assumed that in the future also radio source imaging will play an important role in VGOS observations to monitor source structure. Therefore, an evaluation of different antenna locations based on the uv-coverage of the sources will be of interest as well as source structure effects in general.

The two software packages VieSched++ and VieVS were both modified to provide a fully automatic pipeline to generate large-scale Monte-Carlo simulations. As a consequence, even more and different geometries of the fixed network in combination with the inclusion of twin telescopes can be investigated simply by running the established process based on the base network at that time. Furthermore, this pipeline can also be reused for other types of simulation studies. For example, it can be applied to making decisions of network geometries for the planning of VLBI observing programs within the IVS.

## Data Availability

The datasets used and/or analyzed during the current study are available from the corresponding author on reasonable request.

## Abbreviations

$\omega _{{\text{sky}}}$: Sky-coverage weight factor
$\omega _{{\text{nobs}}}$: Number of observations weight factor
$\omega _{{\text{dur}}}$: Duration weight factor
$\omega _{{\text{idle}}}$: Idle time weight factor
EOP: Earth orientation parameters
GGOS: Global Geodetic Observing System
NNR: No net rotation
NNT: No net translation
NUTX: Nutation in x-direction
NUTY: Nutation in y-direction
RFI: Radio frequency interference
SLR: Satellite laser ranging
UT1: Universal Time
UTC: Coordinated Universal Time
VGOS: VLBI Global Observing System
VieVS: Vienna VLBI and Satellite Software
VLBI: Very Long Baseline Interferometry
XPO: Polar motion in x-direction
YPO: Polar motion in y-direction
